# Audiologic features of elderly with Benign Paroxysmal Positional Vertigo

**DOI:** 10.1016/S1808-8694(15)30794-1

**Published:** 2015-10-19

**Authors:** Nathali Singaretti Moreno, Ana Paula do Rego André

**Affiliations:** 1 Graduation, speech therapist; 2 Doctorate, speech therapist

**Keywords:** hearing, elderly, vertigo

## Abstract

Benign Paroxysmal Positional Vertigo (BPPV) is one of the most common causes of dizziness; it constitutes the most common vestibular disorder in the elderly with vertigo. Its characteristic clinical aspect is dizziness at head movement, with not hearing alteration.

**Aim:**

This paper aims at studying the audiologic characteristics of elderly with BPPV of the posterior semicircular canal.

**Materials and Methods:**

We carried out a retrospective analysis of threshold tonal audiometry exams of 37 senior citizens with posterior semicircular canal BPPV and also of 37 elderly without complaints of dizziness and/or vertigo, and we studied the audiologic characteristics of the two groups.

**Results:**

Both groups had high rates of hearing loss, especially the descending curve sensorineural type, and there was no statistically significant difference between the groups.

**Conclusion:**

Having such data, we can conclude that posterior semicircular canal BPPV has no impact on the hearing loss features of senior citizens; nonetheless, because of the spontaneous degeneration of the vestibulocochlear system, such population has a prevalence of hearing loss.

## INTRODUCTION

Brazil is ceasing to be a country of young people. According to the Instituto Brasileiro de Geografia e Estatística (IBGE or the Brazilian Institute of Geography and Statistics, 2000 census),^1^ the population aged 60 years or above is growing in this country. In 1990, the elderly population was 7.3% of the total population; in 2000 this percentage reached 8.6%. During this period, the number of people aged 60 years or above increased by nearly 4 million individuals. Based on these findings, there is a need for understanding elderly people better. Aging causes communication difficulties, which affect the social relationships of elderly people, and may cause them to have poor self-esteem and self-validation.^2^

Auditory and vestibular vulnerability increases with age, as function deteriorates in these systems.^3^ These degenerative processes are responsible for presbyvertigo and presbyacusis among the geriatric population.^4^, ^5^

Patients describe deafness as a decreased ability to hear, including difficulty to understand speech, intolerance to sound, pressure in the ear, distorted sound sensations, and attention deficit. Hearing loss may be conductive, sensorineural or both, ranging from mild to profound. It is common for elderly patients to become deaf due to inner ear or cochlear nerve injury, which characterizes sensorineural hearing loss.^3^

Sensorineural hypoacusis due to aging is named presbyacusis; it may be caused by noise exposure and senility. In some cases, presbyacusis may be associated with presbyvertigo, which consists of vertigo or dizziness with no apparent cause.^4^

Bodily balance results from stable acceleration between an individual and the surrounding environment, notwithstanding the various accelerations and velocities to which that individual is subjected. Various organs regulate bodily balance (vestibular, system, vision and proprioception), interacting under cerebellar control.^6^

There is a close anatomical relation between auditory sensory organs and bodily balance in the labyrinth. The saccule (part of the vestibular system) is connected to the cochlear duct (part of the auditory system) by the ductus reuniens^7^.

The right and left vestibular systems send information about movement and position of the head to the central nervous system, which is then processed to adjust the body to a new situation. Information provided to the central nervous system needs to be harmonious and precise; any change in such information may alter the status of bodily balance, which might result, for instance in dizziness.^6^

Dizziness may be rotatory (vertigo) or non-rotatory (imbalance, light-headedness, presyncope, etc.). Vertigo is the most common form of dizziness;^3^ it may be present with hearing loss, tinnitus, hypersensitivity to sounds, distortions of sound sensations, difficulty in understanding speech, and auditory attention processing disorders.^6^

### Benign Paroxysmal Positional Vertigo

Benign Paroxysmal Positional Vertigo (BPPV) is one of the most common forms of dizziness.^8^^–^^6^^–^^9^ It is the most common labyrinthic condition in elderly patients with vertigo, about 18% of cases in patients aged over 60 years.^8^

The typical presentation is dizziness or vertigo upon head movements. This clinical picture is usually the result of bending down, looking up, lying down or getting up from bed, and rolling on the bed.^10^, ^11^ Posterior canal BPPV subjects have normal pure tone audiometry tests, and thus have no auditory symptoms.^12^, ^8^

Schuknecht in 1969 described the pathophysiology of cupulolithiasis. This theory states that otoliths become detached from the matrix of the utricle and adhere to the cupula of a semicircular canal, which then stops working as a liner acceleration transducer.^13^ A second theory about the pathophysiology of this condition - canaliculolithiasis - states that the fragments do not adhere to the cupula of a semicircular canal, but rather, float in the endolymph. Head movements would cause these fragments to move and thus stimulate the cupula inappropriately, resulting in vertigo.^14^

This mechanical disease of the peripheral vestibular system affects mainly the posterior semicircular canal, although it may involve other canals simultaneously.^15^, ^16^ It may be associated with vestibular neuritis, Ménière's disease, and others.^17^ In most cases, however, it appears to be idiopathic.^11^ This condition may be found in all age groups, but its incidence increases with age.^10^

Investigation of positional nystagmus aims to define the side and the canal that is involved; it also makes it possible to differentiate canaliculolithiasis from cupulolithiasis. This is important to define which rehabilitation exercises - an essential part of therapy - are appropriate for each case. Vestibular rehabilitation exercises and maneuvers in BPPV are specific for treating each canal.^12^

The purpose of this paper was to verify the audiological features of subjects aged 60 years or more with posterior canal BPPV, since aging may cause degeneration of auditory and vestibular sensory organs.

## MATERIAL AND METHOD

The Research Ethics Committee of the institution approved this study (number 15037/2005).

An analysis was made of 74 medical files of male and female patients aged 60 years or over. The files were provided by the Medical Archival System and separated into groups A and B:

Group A: 37 files of patients with a medical otorhinolaryngological diagnosis of posterior canal BPPV.

Group B: 37 files of patients with no dizziness and/or vertigo.

Inclusion criteria:
Age 60 years or above;
Having undertaken pure tone audiometry;
Intact tympanic membranes.
Exclusion criteria:
Age below 60 years;
No pure tone audiometry;
Presence of tympanic perforation.

All data were analyzed and presented on tables. The SAS 9.0 software was used for the statistical analysis.

Fisher's exact test was applied to analyze any correlation between the presence and absence of hearing loss in both groups and its features (type and configuration of hearing loss).

The significance level as p < 0.05.

## RESULTS

There were 74 files in total, of which 37 files belonged to group A and 37 files to group B.

The mean age of group A patients was 73.3 years, ranging from 62 to 87 years. The mean age of group B patients was 71.7 years, ranging from 60 to 91 years.

Each ear was analyzed individually, given the type and configuration of hearing loss. In group A, we found that there was hearing loss in 70 ears (94.6%); there were 4 normal hearing ears in this group (5.4%). In group B, we found that there was hearing loss in 72 ears (97.3%); there were 2 normal hearing ears in this group (2.7%) (Table 1).

**Table 1:** Frequency of hearing loss / groups.

Group	Hearing loss		Total
	Yes	No	
A	70	4	74
	94.59%	5.41%	
	72	2	
B	97.30%	2.70%	74
Total	142	6	148

p-value = 0,68

The audiometric tests for assessing hearing loss revealed that in group A, 54 ears (76.15%) had sensorineural hearing loss, 5 ears (7.14%) had conductive hearing loss, and 11 ears (15.71%) had mixed hearing loss. In group B, there were 64 ears (88.89%) with sensorineural hearing loss, 1 ear (1.39%) with conductive hearing loss, and 7 ears (9.72%)with mixed hearing loss (Table 2).

**Table 2:** Frequency of the type of hearing loss / groups.

		TYPE		
Group	Conductive	Mixed	Sensorineural	Total
A	5	11	54	
	7.14%	15.71%	76,15%	70
B	1	7	64	
	1.39%	9.72%	88,89%	72
Total	6	18	116	142

p-value = 0,18

The configuration of hearing loss in group A patients was described as follows: there were 40 ears (57.14%) with a descending configuration, 10 ears (14.29%) with U-shaped hearing loss, 5 ears (7.14%) com a flat configuration, and 15 ears (21.43%) with other configurations. In group B, we found 47 ears (65.28%) with a descending configuration, 6 ears (8.33%) with a U-shaped hearing loss, 10 ears (13.89%) with a flat configuration, and 9 ears (12.50%) with other configurations (Table 3).

**Table 3:** Frequency of configuration / group.

		Configuration			Total
Group	Descending	Other configurations	U-shaped	Flat	
A	40	15	10	5	
	57.14%	21.43%	14.29%	7.14%	70
B	47	9	6	10	
	65.28%	12.50%	8.33%	13.89%	72
Total	87	24	16	15	142

p-value = 0,20

## DISCUSSION

Our aim was to analyze the audiological features of elderly patients with posterior canal BPPV, and to compare them with those of elderly patients with no complaints of dizziness and/or vertigo.

We chose to classify hearing loss according to the configuration,^18^ because Davis's classification (1970) according to the mean frequencies at 500, 1 000 and 2 000 Hz would not demonstrate the main feature in our geriatric population, namely that hearing loss occurs at high frequencies.

Aging may cause vertigo and/or dizziness.^5^ Advanced age is a predisposing, aggravating or etiological factor for posterior canal BPPV.^8^

Aging not only affects the vestibular system, but also causes changes in the auditory system; this may lead to sensorineural hypoacusis or presbyacusis.^4^ This degenerative process of the auditory system starts insidiously and progressively; some individuals are unaware of this development as they age.^19^ For this reason, pure tone audiometry is so important for auditory screening in these individuals, regardless of the presence or absence of auditory complaints.

According to Ganança et al.,^8^ the absence of abnormalities in pure tone audiometry and a clinical history of rotating dizziness, vertigo and postural nystagmus, raise the hypothesis of BPPV. Our findings diverge partially from this statement; we found that hearing loss was evenly distributed in both groups (with both present and absent posterior canal BPPV). We concluded that hearing in elderly individuals with posterior canal BPPV is altered due to age itself.

Sensorineural descending hearing loss is prevalent in elderly individuals; the threshold is decreased as age progresses.^20^ We found this same prevalence of hearing loss in both groups, showing that posterior canal BPPV does not alter the features of hearing loss in elderly individuals.

In Another study, 97.1% of a sample of elderly individuals with complaints of dizziness had sensorineural hearing loss, and 81.8% of this sample had descending hearing loss. This study also showed that the prevailing vestibular complaint was postural vertigo (a feature of posterior canal BPPV), which was encountered in 61.8% of the sample.^21^

Inherent degenerative processes may explain this type of hearing loss and the prevalence of BPPV in elderly individuals.

We concluded in this study that we may not state with certainty that elderly individuals with a diagnosis of posterior canal BPPV have no auditory alterations, since the study sample was a geriatric population, in which degenerative processes that may alter the auditory and/or vestibular system - such as hearing loss and posterior canal BPPV - may occur.

## CONCLUSION

We concluded in this study that posterior canal BPPV does not affect the features of hearing loss in elderly subjects. We were unable to state, however, that elderly individuals with posterior canal BPPV have no hearing loss, since spontaneous degeneration of the vestibulocochlear system is inherent to all individuals aged 60 years or above.
